# Vitamin D for inflammation biomarkers in coronary artery disease: A protocol for systematic review and meta-analysis

**DOI:** 10.1097/MD.0000000000021407

**Published:** 2020-07-31

**Authors:** Yiru Wang, Yifan Zhang, Jing Wei, Wenting Du, Jie Ding, Yiyi Zhang, Na Zhang, Meijiao Mao, Ping Liu

**Affiliations:** a Longhua Hospital affiliated to Shanghai University of Traditional Chinese Medicine; b Department of Traditional Chinese Medicine, Shanghai Xuhui Central Hospital, Shanghai, China.

**Keywords:** vitamin D, inflammation, coronary artery disease, review, meta-analysis

## Abstract

**Background:**

Coronary artery disease (CAD) is a clinically common coronary heart disease. Vitamin D might be beneficial in CAD patients through its favorable effects on inflammation biomarkers. This study will be performed to examine the effects of Vitamin D supplementation on inflammatory markers in CAD patients.

**Methods:**

We will search the electronical databases and hand-searching journals or reference lists. The study screening and data extraction will be carried out by 2 investigators independently. The primary outcome is inflammatory biomarkers of peripheral blood. Secondary outcomes are triglyceride, total cholesterol, high-density lipoprotein cholesterol levels, low-density lipoprotein cholesterol, and blood pressure. Review Manager (RevMan, version 5.3; The Nordic Cochrane Centre, Copenhagen, Denmark: http://community.cochrane.org) V.5.3 software will be used to compute the data.

**Results:**

The results of the study will provide a reliable evidence to assess the efficacy of Vitamin D supplement on inflammation biomarkers of CAD patients.

**Conclusion:**

The conclusion of our systematic review will answer whether Vitamin D is an effective intervention to relieve inflammation of CAD patients.

**Ethics:**

Because all of the data used in this review has been published, this review does not require ethical approval.

**Registration number:**

INPLASY202060072.

## 1 Introduction

Cardiovascular disease is one of the leading death causes in the worldwide population, with a prevalence of 48% among people over 20 years old.^[1,2]^ Coronary artery disease (CAD) is the leading cause of death in the United States.^[3]^ CAD was previously regarded as a lipid accumulation–intermediated illness,^[4]^ but now it has been evidently shown to involve a continuous inflammatory response in the all stages of CAD.^[1]^ The relationship between traditional cardiovascular risk factors and inflammation is more complicated and closer than formerly thought.^[5]^

Vitamin D (VD) is obtaining growing attention for its original association with CAD.^[6,7]^ Some studies have reported that CAD patients have lower circulating concentrations of VD than health people.^[8,9]^ VD is a negative regulator of inflammation^[10]^ and plays an crucial role in decreasing inflammation biomarkers.^[11]^ Given the anti-inflammatory effects of VD, it realized us logically to consider whether VD supplementation protects against CAD by reducing the inflammatory biomarkers levels. The effects of VD supplementation on CAD prevention and treatment have not been clearly concluded. Increased food-generated VD may protect against intermediate and hard cardiovascular endpoints,^[12]^ but according to another report VD did not lessen main CAD events or other cardiovascular end points.^[13]^ However, the sample size of these studies was small, the methodological quality differed from low to high, and the results were inconsonant.

Therefore, we intend to use meta-analysis method, search global clinical research about VD on inflammation biomarkers of CAD patients, systematically evaluate the effects of VD treatment on CAD, and provide more scientific evidence for clinical strategy. In this meta-analysis protocol, inflammatory biomarkers are selected as the major outcome.

## 2 Methods

This systematic review protocol has been registered on INPLASY202060072 (DOI number: 10.37766/inplasy2020.6.0072; website: https://inplasy.com/inplasy-2020-6-0072/). The protocol follows the Cochrane Handbook for Systematic Reviews of Interventions and the Preferred Reporting Items for Systematic Reviews and Meta-Analysis Protocol (PRISMA-P) statement guidelines.^[14]^ We will describe the changes in our full review if needed.

### 2.1 Inclusion criteria for study selection

#### 2.1.1 Participants

All patients must meet the CAD diagnostic criteria established by the American College of Cardiology/ American Heart Association.^[15]^ There will be no limitations of countries, ages, gender, and comorbidity. Patients with acute myocardial infarction, myocarditis, uncontrolled chronic diseases, mental illness, or related drug allergy will be excluded. Additionally, patients who consume VD supplements within the past 3 months will also be excluded. will be excluded.

#### 2.1.2 Interventions

Patients in the experimental group should be treated with VD or VD plus conventional medicine, such as statin, aspirin, β-blockers, et al. The patients in control group should be treated with VD placebo or VD placebo plus the same conventional medicine. It will not be limited to VD doses and courses.

#### 2.1.3 Outcomes

Primary outcomes. Inflammation biomarkers of peripheral blood include CRP, IL-2, IL-6, and IL-10.
Secondary outcomes. Triglyceride, total cholesterol, high-density lipoprotein cholesterol levels, low-density lipoprotein cholesterol, blood pressure, and adverse effects.

#### 2.1.4 Types of studies

All the clinical randomized controlled trials of VD for CAD will be included in the review.

### 2.2 Search methods for identifying the studies

#### 2.2.1 Search sources

We will search the following electronic databases, including PubMed, Cochrane Library, Embase, Web of Science, China National Knowledge Infrastructure, Chinese Biological and Medical database, VIP, and Wanfang Database. The search time limit is from the database inception to June 2020. In addition, we will also manually search for the relevant journals, conference articles, dissertations, and unpublished researches to avoid missing grey literature. Two investigators (YFZ and JW) will search for the potential studies according to the methods independently.

The following search strategy will be used in PubMed:

1. 1 Vitamin D [mh]
2. (Deficiency, Vitamin D OR Vitamin D Deficiencies OR Deficiencies, Vitamin D) [tw]
3. 1 OR 2
4. Coronary artery disease [mh]
5. Coronary artery diseases [mh]
6. (Arteriosclerosis, Coronary OR Coronary Arteriosclerosis OR Coronary Atherosclerosis OR Atherosclerosis, Coronary OR Atherosclerosis, Coronary OR Arteriosclerosis, Coronary OR Coronary Atherosclerosis OR Coronary Arteriosclerosis OR Artery Disease, Coronary OR Diseases, Coronary Artery OR Coronary Artery Diseases OR Artery Diseases, Coronary OR Disease, Coronary Artery) [tw]
7. OR 5 OR 6
8. Inflammation [mh]
9. (Inflammations OR Inflammatory Response, Innate OR Innate Inflammatory Response OR Innate Inflammatory Responses) [tw]
10. Inflammatory [tw]
11. 8 OR 9 OR 10
12. Biomarker [mh]
13. (Markers, Laboratory OR Laboratory Markers OR Marker, Laboratory OR Laboratory Marker OR Marker, Serum OR Markers, Serum OR Serum Markers OR Serum Marker OR Marker, Viral OR Viral Marker OR Markers, Viral OR Viral Markers OR Biochemical Marker OR Marker, Biochemical OR Biochemical Markers OR Markers, Biochemical OR Markers, Biologic OR Biologic Markers OR Marker, Biologic OR Markers, Biological OR Marker, Biological OR Biologic Marker OR Biological Marker OR Biological Markers OR Marker, Surrogate OR Surrogate Marker OR Surrogate Markers OR Markers, Surrogate OR Endpoints, Surrogate OR Surrogate Endpoints OR End Point, Surrogate OR Surrogate Endpoint OR Surrogate End Point OR Endpoint, Surrogate OR End Points, Surrogate OR Surrogate End Points OR Clinical Marker OR Clinical Markers OR Marker, Clinical OR Markers, Clinical OR Marker, Immunologic OR Marker, Immune OR Markers, Immune OR Immunologic Marker OR Immune Marker OR Immune Markers OR Markers, Immunologic OR Immunologic Markers) [tw]
14. 12 OR 13
15. AND 7 AND 11 AND 14
16. randomized controlled trial [pt]
17. controlled clinical trial [pt]
18. randomized [tiab]
19. human trials as topic [mesh: noexp]
20. randomly [tiab]
21. trial [ti]
22. 16 OR 17 OR 18 OR 19 OR 20 OR 21
23. humans [mh] NOT animals [mh]
24. 22 and 23
25. 15 and 24

Pubmed search syntax

[mh] denotes a Medical Subject Heading (Mesh) term (‘exploded’)

[tw] denotes text word

[pt] denotes a Publication Type term

[tiab] denotes a word in the title OR Abstract

[sh] denotes a subheading

[mesh: noexp] denotes a Medical Subject Heading (Mesh) term (not ‘exploded’)

[ti] denotes a word in the title.

#### 2.2.2 Search strategies.

**Figure 1:**
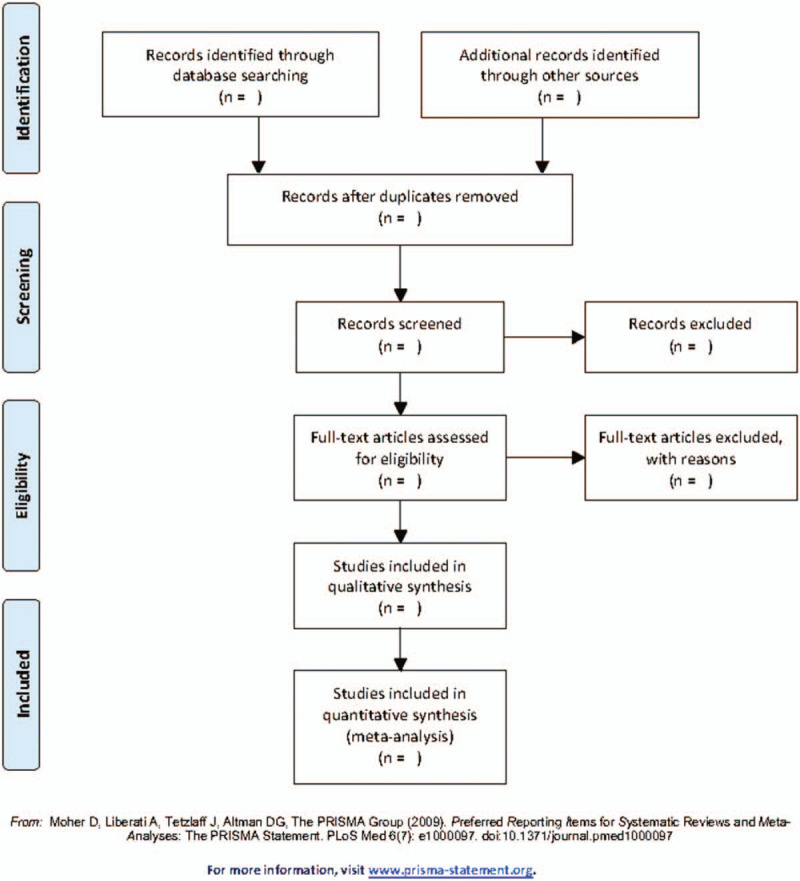
Flow diagram of study selection process.

All the searched studies will be exported to the Endnote software (version 9.3.1, Thomas Reuters, CA) and duplicates will be excluded. The first selection will contain scanning of the titles and abstracts of the retrieved studies by 2 independent investigators (WTD and JD). Then the same independent authors will read the full text to decide if the article should be included. Finally, a third author (YYZ) will check whether the results are same. If there are any disagreements, YYZ will resolve these through discussing with the above 2 authors and made the final decision. The procedure of studies selection is presented in a flow diagram (Fig. 1).

### 2.3 Data extraction and synthesis

Two researchers (NZ and MJM) will independently use Review Manager (Revman, version 5.3.5, Copenhagen: The Nordic Cochrane Centre, The Cochrane Collaboration, 2014) software to extract literature data. A third research member (PL) will detect data consistency and check the final database. If the data of included study are uncertain, lost or in the form that not extractable, we will email the authors of the study to request for an affirmation.

The data extraction contents are as follows:

Common information: title, first author, corresponding author, publishing time and contact information.
Study designs: article type, sample size, baseline balance, randomized method, blinding method, data loss and analysis, and selective reporting.
Participants: region, ethnicity, age, male/female ratio, diagnostic criteria, combined disease, and duration of illness.
Intervention: treatment period, treatment frequency, drug dosage, combined drugs, details about the control group and follow-up.
Outcomes: main observation indicators (inflammation biomarkers of peripheral blood include CRP, IL-2, IL-6, and IL-10) and secondary observation indicators (triglyceride, total cholesterol, high-density lipoprotein cholesterol levels, low-density lipoprotein cholesterol and blood pressure) and adverse effects.
Others: sources of funding, ethics audit.

### 2.4 Risk of bias assessment

Two independent reviewers (NZ and MJM) will use the Cochrane Collaboration's tool to assess the methodological quality and risk of bias. The assessment contains 7 dimensions: sequence generation; allocation concealment; blinding of participants and personnel; blinding of outcome assessors; incomplete outcome data; selective outcome reporting; and other issues. Each bias divides into low, unclear, and high level according to the Cochrane Handbook for Systematic Reviews of Interventions (Version 5.3). The inconsistencies cannot be resolved in this review will search consensus for a third author (PL) as required. Or else, we will consult with the Cochrane Professional Group for final resolution.

### 2.5 Assessment of heterogeneity

We will use Chi-squared test to conclude the homogeneity of included studies. I^2^ statistic > 50% suggests significant heterogeneity of the test (using a random effect model), and I^2^ statistic < 50% suggests that there is no statistical heterogeneity or heterogeneity is small relatively (using a fixed effect model).

If there is significant clinical heterogeneity, firstly we will check the raw data in the studies. Next, the sensitivity analysis or subgroup analysis will be used to find the cause of heterogeneity. If it is unable to judge the source of heterogeneity, we will choose descriptive analysis.

We will perform subgroup analysis to measure the heterogeneity of the researches as following reasons:

(1) Clinical consideration:
  different age, sex, and race
  different VD dosage and VD form
  different treatment course
(2) Methodology consideration: tests with unclear or high risks of bias

### 2.6 Assessment of reporting bias

When a result of a meta-analysis includes more than ten articles, funnel plots will be used to determine the risk of publication bias. If the 2 sides are symmetrical, there is no obvious publication bias. If the image is uncertain, we will use STATA (version 12.0; Texas, USA: https://www.stata.com/) 12.0 software to perform Egger test for quantitative analysis.

### 2.7 Sensitivity analysis

If possible, low-quality studies are included, we will perform the sensitivity analysis. This is a key method mainly used to evaluate the robustness and reliability of the combined meta-analysis results. The method is eliminating each included article, or changing the inclusion and exclusion criteria or removing some types of articles.

### 2.8 Grading the quality of evidence

GRADE profiler software Version 3.6 (GRADEpro GDT, McMaster University, 2015; Evidence Prime, Inc, https://gradepro.org/) will be used to evaluate the quality of evidence. The grades will be divided into 4 levels: high, medium, low, and extremely low.

## 3 Discussion

To the best known of our knowledge, this systematic review and meta-analysis is the first to measure the effect of VD on inflammation biomarkers of CAD patients. Some clinical studies found that VD could effectively treat cardiovascular disease and has been used in some clinical practice.^[16,17]^ But there is no systematic review regarding its effect and safety so far. Therefore, we will perform this high-quality research with the aim of providing a vigorous proof of effectiveness and safety for clinical strategy and health policymakers.

However, there may be some potential limitations in this systematic review. For example, different doses and frequencies of VD may result in significant heterogeneity and poor methodological quality. We will only include articles published in Chinese or English, which may increase the bias. In addition, different nationalities and ages of the patients also may be a heterogeneity risk.

## Author contributions

YRW and PL contributed to the conception of the study. The manuscript protocol was drafted by YRW and was revised by PL. The search strategy was developed by all the authors and will be performed by YFZ and JW. WTD and JD will independently screen the potential studies. NZ and MJM will extract data from the include studies and assess the risk of bias. YRW will complete the data synthesis, assessment of reporting bias, sensitivity analysis and the quality of evidence. PL and YYZ will arbitrate in cases of disagreement and ensure the absence of errors. All authors approved the publication of the protocol.

**Conceptualization:** YRW, PL.

**Formal analysis:** YRW and JW.

**Software:** YRW and YFZ.

**Supervision:** PL.

**Writing – original draft:** YRW.

**Writing – review & editing:** PL.
